# Integration of miRNA and mRNA Co-Expression Reveals Potential Regulatory Roles of miRNAs in Developmental and Immunological Processes in Calf Ileum during Early Growth

**DOI:** 10.3390/cells7090134

**Published:** 2018-09-11

**Authors:** Duy N. Do, Pier-Luc Dudemaine, Bridget E. Fomenky, Eveline M. Ibeagha-Awemu

**Affiliations:** 1 Agriculture and Agri-Food Canada, Sherbrooke Research and Development Centre, Sherbrooke, QC J1M 0C8, Canada; DuyNgoc.Do@AGR.GC.CA (D.N.D.); Pier-Luc.Dudemaine@AGR.GC.CA (P.-L.D.); bridget.fomenky.1@ulaval.ca (B.E.F.); 2 Department of Animal Science, McGill University, Ste-Anne-de-Bellevue, QC H9X 3V9, Canada; 3 Département de Sciences Animale, Université Laval, Quebec, QC G1V 0A6, Canada

**Keywords:** calf, Ileum, miRNA-mRNA integration, miRNA sequencing, growth, development

## Abstract

This study aimed to investigate the potential regulatory roles of miRNAs in calf ileum developmental transition from the pre- to the post-weaning period. For this purpose, ileum tissues were collected from eight calves at the pre-weaning period and another eight calves at the post-weaning period and miRNA expression characterized by miRNA sequencing, followed by functional analyses. A total of 388 miRNAs, including 81 novel miRNAs, were identified. A total of 220 miRNAs were differentially expressed (DE) between the two periods. The potential functions of DE miRNAs in ileum development were supported by significant enrichment of their target genes in gene ontology terms related to metabolic processes and transcription factor activities or pathways related to metabolism (peroxisomes), vitamin digestion and absorption, lipid and protein metabolism, as well as intracellular signaling. Integration of DE miRNAs and DE mRNAs revealed several DE miRNA-mRNA pairs with crucial roles in ileum development (bta-miR-374a—*FBXO18,* bta-miR-374a—*GTPBP3*, bta-miR-374a—*GNB2*) and immune function (bta-miR-15b—*IKBKB*). This is the first integrated miRNA-mRNA analysis exploring the potential roles of miRNAs in calf ileum growth and development during early life.

## 1. Introduction

MicroRNAs (miRNAs) are small (~22 nucleotides) endogenous RNA molecules that regulate gene expression post-transcriptionally by targeting principally the 3′ untranslated region (3′UTR) of genes and to some extend the 5′UTR, introns and coding region of mRNAs [1]. They play key roles in a wide range of biological processes [2]. In bovine, miRNAs have been shown to play important roles in embryonic development [3,4,5], mammary gland [6,7] and adipose tissue [8] functions and also in the regulation of production traits such as milk yield [6,9,10], milk quality [11] and diseases like mastitis [12,13] and Johne’s disease [14]. The importance of miRNAs in gut development and disease (mostly inflammatory bowel disease) has been extensively studied in humans [15,16,17,18]. For instance, several miRNAs have been reported to be relevant for different aspects of inflammatory bowel disease (IBD), including miRNAs important for intestinal fibrosis (miR-29, miR-200b, miR-21, miR-192), epithelial barrier and immune function in IBD pathogenesis (miR-192, miR-21, miR-126, miR-155, miR-106a) [19].

Data from a few studies suggest important roles for miRNAs in calf’s early life [20,21]. For instance, Liang et al. (2016a) [21] identified several dominantly expressed miRNAs (miR-143 (30% of read counts), miR-192 (15%), miR-10a (12%) and miR-10b (8%)) in ileum tissues of dairy calves collected at 30 min after birth and at 7, 21 and 42 days old. Furthermore, several temporally expressed miRNAs (miR-146, miR-191, miR-33, miR-7, miR-99/100, miR-486, miR-145, miR-196 and miR-211), regional specific miRNAs (miR-192/215, miR-194, miR-196, miR-205 and miR-31) and miRNAs (miR-15/16, miR-29 and miR-196) linked to bacterial abundance in the jejunum and ileum were also reported [21]. Moreover, several ileum miRNAs are reported to play important roles in host responses to *Mycobacterium avium* subspecies paratuberculosis infection, such as the role of bta-miR-196b in the proliferation of endothelial cells and bta-miR-146b in bacteria recognition and regulation of the inflammatory response [22].

During early life, calves undergo major physiological and digestive changes, including adaptation to diet changes from pre- to post-weaning. The interactions between transcriptional and post-transcriptional mechanisms are known to coordinate these developmental transitions via regulation of gene expression. Recently, we characterized the long non-coding RNA (lncRNA) expression in ileum tissues of calves and functional inference of identified lncRNA (623 known and 1505 novel) *cis* target genes revealed potential roles in growth and development as well as in posttranscriptional gene silencing by RNA or miRNA processing processes and in disease resistance mechanisms [23]. Moreover, we also observed that 122 miRNAs were significantly differentially expressed between the pre- and post-weaning periods in the rumen, suggesting important roles of miRNAs in calf gut during early life [24]. Therefore, we hypothesize that miRNAs might play important roles in ileum development during the pre- and post-weaning periods. Thus, in the current study, we performed integrated miRNA-mRNA co-expression analyses to uncover the potential roles of miRNAs in ileum development at the pre- and post-weaning periods.

## 2. Materials and Methods

### 2.1. Animals and Management

Procedures for animal management were conducted according to the Canadian national codes of practice for the care and handling of farm animals (http://www.nfacc.ca/codes-of-practice) and approved by the animal care and ethics committee of Agriculture and Agri-Food Canada (CIPA #442).

Experimental details have been described in our previous studies [23,24]. Briefly, sixteen 2–7 days-old Holstein calves were raised for a period of 96 days in individual pens. In the first week of the experiment, calves were fed milk replacer (6 L/day for the first four days and 9 L/day thereafter, Goliath XLR 27-16, La Coop, Montreal, QC, Canada), and then starter feed (Munerie Sawyerville Inc., Cookshire-Eaton, QC, Canada) was introduced (ad libitum) from the second week. After weaning, calves were fed with starter feed and hay ad libitum. The calves were weighed weekly until euthanization. At experiment D33 (pre-weaning), eight calves were humanely euthanized and another eight calves on D96 (post-weaning), for the collection of ileum tissue samples. Tissues were aseptically collected, snap frozen in liquid nitrogen, and stored at −80 °C until used.

### 2.2. Total RNA Purification

Ileum tissue (30 mg/sample) was used for total RNA isolation using miRNeasy Kit (Qiagen Inc., Toronto, ON, Canada). Potentially contaminating genomic DNA was removed by treating 10 µg of purified RNA (10 µg) with DNase (Turbo DNA-free™ Kit, Ambion Inc., Foster City, CA, USA). The RNA concentration (before and after digestion) and its quality (integrity) after DNase treatment were assessed with Nanodrop ND-1000 (NanoDrop Technologies, Wilmington, DE, USA) and Agilent 2100 Bioanalyzer (Agilent Technologies, Santa Clara, CA, USA), respectively. The RNA integrity number (RIN) of all samples was greater than 8 and a small RNA peak area was visible on the electropherogram [25].

### 2.3. miRNA Library Preparation and Sequencing

Libraries (*n* = 16) were prepared and barcoded for sequencing according to Do et al. [10]. Briefly, polyacrylamide gel electrophoresis was used to size separate miRNA libraries from other RNA species. An elution buffer (10 mM Tris-HCl pH 7.5; 50 mM NaCl, 1 mM EDTA) was used to elute the libraries from the gel. Eluted library was concentrated using DNA clean and concentrator-5 (Zymo Research, Irvine, CA, USA). The concentration of purified libraries was evaluated using Picogreen assay (Life Technologies, Waltham, MA, USA) and a Nanodrop 3300 fluorescent spectrophotometer (NanoDrop Technologies), and further confirmed by qPCR using the Kapa Library Quantification Kit for Illumina platforms (KAPA Biosystems, Wilmington, MA, USA). Libraries were multiplexed in equimolar concentrations and sequenced in one lane on an Illumina HiSeq 2500 platform following Illumina’s recommended protocol to generate single end data of 50-bases by The Centre for Applied Genomics, The Hospital for Sick Children, Toronto, Canada (http://www.tcag.ca/).

### 2.4. Small RNA Sequence Data Analysis

Bioinformatics processing of generated small RNA sequences was done as previously described [10,24]. Briefly, the raw sequence data (16 fastq files) was checked for sequencing quality with FastQC program v0.11.3 (http://www.bioinformatics.babraham.ac.uk/projects/fastqc/). Trimming of 3′ and 5′ adaptor sequences, contaminants and repeats was accomplished with Cutadapt v1.2.2 (https://cutadapt.readthedocs.org/). Then, FASTQ Quality Filter tool of FASTX-toolkit (http://hannonlab.cshl.edu/fastx_toolkit/) was used to remove reads having a Phred score <20 for at least 50% of the bases and reads shorter than 18 nucleotides or longer than 30 nucleotides. Clean reads that passed all filtering criteria from the 16 files were parsed into one file and mapped to the bovine genome (UMD3.1) using bowtie 1.0.0 (http://bowtie-bio.sourceforge.net/index.shtml) [26]. Reads that mapped to other RNA species (rRNA, tRNA, snRNA and snoRNA) in the Rfam RNA family database (http://rfam.xfam.org/) or to more than five positions of the genome were removed.

### 2.5. Identification of Known miRNA and Novel miRNA Discovery

The identification of known miRNAs was performed with miRBase v21 http://www.mirbase.org/) (Kozomara and Griffiths-Jones, 2014), while novel miRNA discovery was achieved with miRDeep2 v2.0.0.8 (https://github.com/rajewsky-lab/mirdeep2) [27]. MiRDeep2 was designed to detect miRNAs from deep sequence reads using a probabilistic algorithm based on the miRNA biogenesis model. The core and quantifier modules of miRDeep2 were applied to discover novel miRNAs in the pooled dataset of all the libraries while the quantifier module was used to profile the detected miRNAs in each library. MiRDeep2 score higher than five was used as cuff point for the identification of novel miRNAs. Subsequently, a threshold of 10 counts per million and present in ≥2 libraries was applied to remove lowly expressed miRNAs. MiRNAs meeting these criteria were further used in downstream analyses including differential expression (DE) analysis.

### 2.6. Differential miRNA Expression

DeSeq2 (v1.14.1) (https://bioconductor.org/packages/release/bioc/html/DESeq2.html) [28], which implements a negative binomial model, was used to perform differential miRNA expression analysis. Following normalization, normalized counts of miRNAs at D96 were compared with corresponding values on D33. Significant differential miRNA expression between D33 (pre-weaning) and D96 (post-weaning) was defined as having a Benjamini and Hochberg [29] false discovery rate (FDR) or corrected *p*-value < 0.05.

### 2.7. Predicted Target Genes of miRNAs

In order to investigate the functions of the most highly expressed miRNAs and differently expressed (DE) miRNAs, we firstly predicted their target mRNAs. Perl scripts (targetscan_60.pl and targetscan_61_context_scores.pl) (http://targetscan.org) were used to predict target mRNAs and to calculate their context scores, respectively. Predicted target mRNAs with context + scores above 95th percentile were further used [9,10,30]. The predicted target mRNAs were then filtered against the mRNA transcriptome obtained from ileum tissues of the same animals. Only predicted target genes that were expressed in the mRNA transcriptome of the ileum tissues of the animals [23] were retained for further analysis.

### 2.8. miRNA–mRNA Co-Expression Analysis and Target Gene Enrichment

For miRNA–mRNA co-expression, the Pearson correlation coefficient between target mRNAs (retained above) and DE miRNAs were calculated. A miRNA-mRNA pair was considered co-expressed if it had a negative and significant correlation value at FDR < 0.05. The mRNAs significantly correlated with miRNAs were then used for downstream target gene ontology and KEGG pathways enrichment using ClueGO (http://apps.cytoscape.org/apps/cluego) [31]. For ClueGO analysis, a hypergeometric test was used for enrichment analyses and Benjamini–Hochberg [29] correction was used for multiple testing correction (FDR < 0.05). Since KEGG pathways enrichment relied on the human database (due to lack of information in bovine), we used a less stringent threshold (uncorrected *p*-value < 0.05) to declare if a pathway was significantly enriched. Interactions between miRNAs and mRNAs were visualized with Cytoscape (http://www.cytoscape.org/) [32].

### 2.9. Real-Time Quantitative PCR

The method of real-time quantitative PCR was used to validate the expression of four DE (bta-miR-142-5p, miR-146a, miR-24-3p and miR-374b) and two non-DE (bta-miR-486-5p and miR-193b) miRNAs. The same total RNA used in miRNA-sequencing was used. Total RNA was reverse transcribed with Universal cDNA Synthesis Kit II from Exiqon (Exiqon Inc., Woburn, MA, USA), following the manufacturer’s instructions. ExiLENT SYBR^®^ Green Master Mix Kit (Exiqon, Woburn, MA, USA) and the miRCURY LNA™ Assay (Exiqon, Woburn, MA, USA) specific for each miRNA listed above were used to perform Quantitative qPCR on a StepOne Plus System (Applied Biosystems, Foster City, CA, USA) according to the manufacturer’s instructions. Bta-miR-126-3p was used as endogenous control to assess the expression level of miRNAs using the comparative Ct (ΔΔCt) method. Bta-miR-126-3p was selected as an endogenous control based on its consistent expression throughout all the analyzed samples on D33 and D96.

## 3. Results

### 3.1. Identification and Characterization of Ileum miRNAs

MiRNA sequencing of 16 libraries generated a total of 185,458,022 reads. After adaptor trimming, size selection and quality filtering, 150,999,506 (81.4%) reads with length ranging from 18 to 30 nucleotides and having a phred score >20 were retained for analysis (Table S1). Out of this number, 133,698,161 reads (88.5%) mapped to unique positions on the bovine genome (University of Maryland assembly of *B. taurus*, release 3.1; UMD.3.1), 10,661,520 (7.1%) were unmapped, while 1,150,263 (0.8%) mapped to more than five positions and were discarded (Table S1). Mapped reads belonging to other RNA species, tRNA (3,153,316 (2.1%)), rRNA (480,099 (0.3%)), snRNA (236,118 (0.2%)) and snoRNA (1,620,029 (1.1%)) were discarded. The majority of miRNAs retained for further analyses were 22 nucleotides long (Table S2).

Novel miRNAs were considered to have a minimum MiRDeep2 score of five, as shown in Table S3. After removing lowly expressed reads, a total of 307 known and 81 novel miRNAs satisfying the conditions of having at least 10 read counts per million and present in a minimum of two libraries were used for DE analysis (Table S4a,b).

Abundantly expressed miRNAs having >3% of the total read counts on D33 and D96 were bta-miR-143, bta-miR-192, bta-miR-26a and bta-miR-21-5p, while bta-miR-191, bta-miR-10b, bta-miR-148a and bta-miR-10a were highly expressed with >3% of total read counts on D96 (post-weaning) only (Table 1). The 20 commonly highly expressed miRNAs (>1% of total read counts) targeted 2609 unique genes (Table 1 and Table S5a). The target genes were significantly enriched in 459 biological processes (BP), 53 cellular components (CC) and 43 molecular function (MF) gene ontology (GO) terms (Table S5b), as well as in 14 KEGG pathways (Table S5c). Single-organism developmental process (FDR = 1.13 × 10^−10^), intracellular (FDR = 4.63 × 10^−17^), and protein binding (FDR = 1.10 × 10^−5^) were the most significantly enriched BP, CC and MF GO terms, respectively (Table 2), while MAPK signaling pathway was the most significantly enriched KEGG pathway (Table 3). Moreover, a novel miRNA, bta-miR-22-24033, was the most highly expressed among novel miRNAs (accounted for 0.3% of total read counts) (Table S4b).

### 3.2. Differentially Expressed miRNAs and Downstream Target Gene Enrichment Analyses

**Figure 1:**
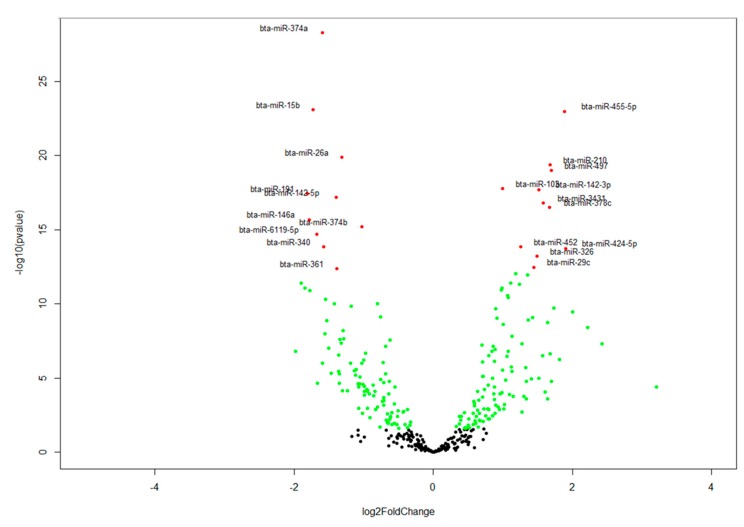
Volcano plot depicting miRNA differential expression results. Each dot represents a miRNA. Green and red dots represent miRNAs significantly differentially expressed at FDR < 0.05 and FDR < 1 × 10^11^, respectively. Black dots represent miRNAs that were not differentially expressed. Differential expression analysis was accomplished with DeSeq2.

A total of 220 miRNAs (104 up-regulated and 116 down-regulated) were significantly DE between D33 (pre-weaning) and D96 (post-weaning) (Figure 1, Table S6a). Bta-miR-374a (FDR = 5.00 × 10^29^), bta-miR-15b (FDR = 7.96 × 10^24^) and bta-miR-26a (FDR = 1.30 × 10^20^) were the most significantly down-regulated miRNAs, while bta-miR-455-5p (FDR = 1.01 × 10^23^), bta-miR-210 (FDR = 4.23 × 10^20^) and bta-miR-497 (FDR = 9.95 × 10^20^) were the most significantly up-regulated miRNAs (Table 4).

**Figure 2:**
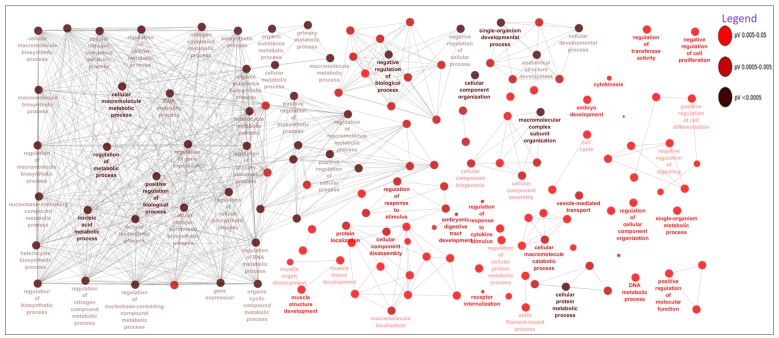
The ClueGO results for biological processes gene ontology terms enrichment for target genes (mRNAs) of differentially expressed miRNAs and relationships between them. The nodes (round shape) represent gene ontology terms, node color represents the level of significance as indicated in the legend, while node size reflects the number of genes enriched in each gene ontology term.

**Figure 3:**
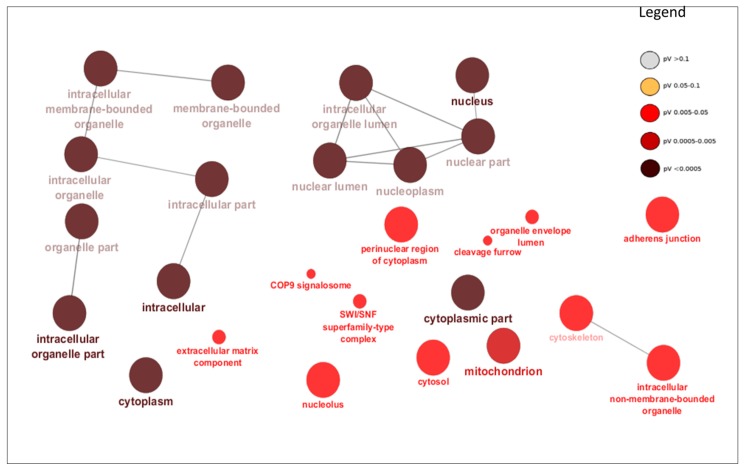
The ClueGO results for cellular processes gene ontology terms enrichment for target genes of differentially expressed miRNAs and relationships between them. The nodes (round shape) represent gene ontology terms, node color represents the level of significance as indicated in the legend, while node size reflects the number of genes enriched in each gene ontology term.

**Figure 4:**
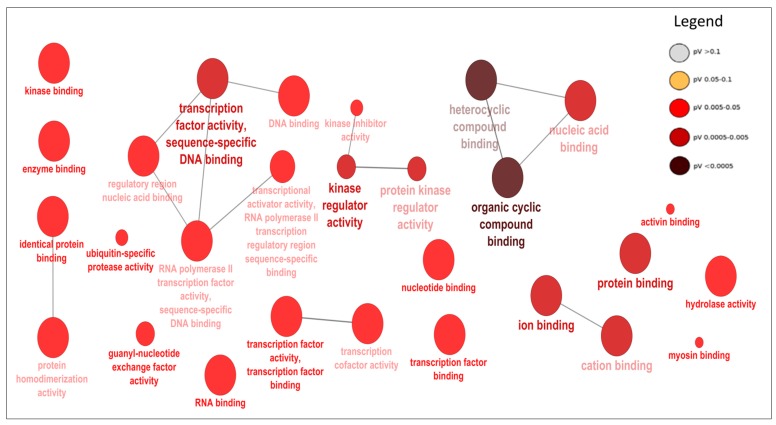
The ClueGO results for molecular functions gene ontology terms enrichment for target genes of differentially expressed miRNAs and relationships between them. The nodes (round shape) represent gene ontology terms, node color represents the level of significance as indicated in the legend, while node size reflects the number of genes enriched in each gene ontology term.

**Figure 5:**
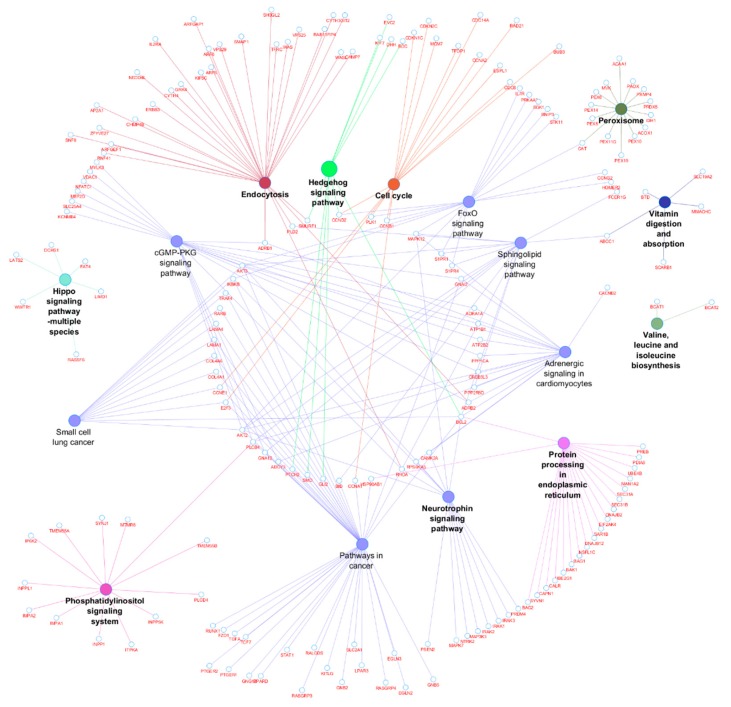
The ClueGO results for KEGG pathways enrichment for target genes of differentially expressed miRNAs and relationships between them. The nodes (round shapes) represent KEGG pathways or genes enriched in the pathways.

**Figure 6:**
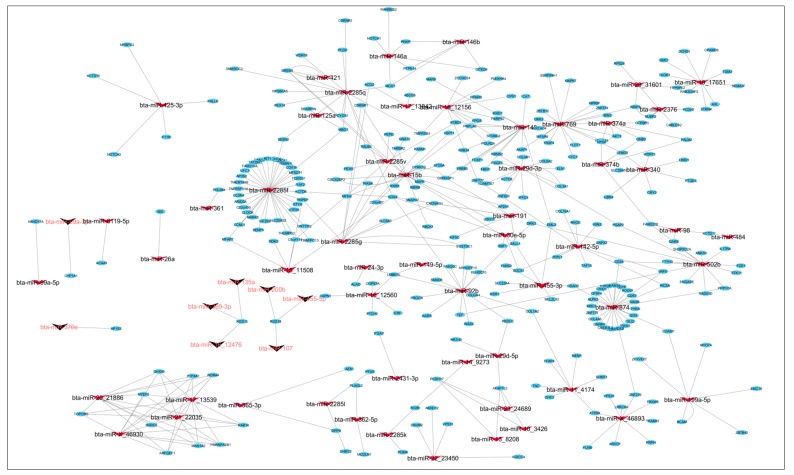
The Cytoscape visualization of the relationships between differentially expressed miRNAs and their target mRNAs. The nodes present either genes (round shape) or miRNAs (V shape). The up- and down-regulated miRNAs are colored red and black, respectively.

The DE miRNAs (220 miRNAs) were predicted to target 11,691 mRNAs (Table S6b). Using mRNA transcriptome data of the same samples, 1560 mRNAs out of the predicted 11,691 mRNAs, were significantly and negatively correlated with their targeting miRNAs (Table S6c). Bta-miR-2285f had the highest number of target genes (172), while *AGO2* gene was the most popular target for DE miRNAs (targeted by 25 DE miRNAs) (Table S6c). Other common target genes for DE miRNAs were *SLC25A46*, *KCTD13* and *PAXIP1,* each targeted by 9 DE miRNAs (Table S6c). The GO enrichment analyses of the 1560 target genes (significantly and negatively correlated with miRNA) indicated that 158, 26 and 28 of them were significantly enriched in BP-, CC- and MF-GO terms, respectively (Table S7a–c). The most enriched BP-, CC- and MF-GO terms were cellular macromolecule metabolic process (FDR = 9.38 × 10^10^), intracellular (FDR = 3.37 × 10^19^) and organic cyclic compound binding (FDR = 1.19 × 10^4^), respectively (Table 5, Figure 2, Figure 3 and Figure 4). Moreover, 16 KEGG pathways were significantly enriched for the target genes (1560) of 220 DE miRNAs, and peroxisome (*p* = 0.004) and Hedgehog signaling pathways (*p* = 0.006) were the most significantly enriched (Table S7d, Figure 5). Moreover, among the 1560 target genes negatively correlated with miRNAs, 278 were also significantly DE between D33 and D96 in our previous study (Table S8) [23]. The 278 genes were the targets for 64 DE miRNAs. SOX4 was the most common target, since it was targeted by 6 different miRNAs (bta-miR-191, bta-miR-30e-5p, bta-miR-15-11508, bta-miR-2285f, bta-miR-92b and bta-miR-2285q). Meanwhile, bta-miR-2285f and bta-miR-874 had the highest number of target genes (37 and 28, respectively) (Figure 6).

### 3.3. Real Time Quantitative PCR Validation 

**Figure 7:**
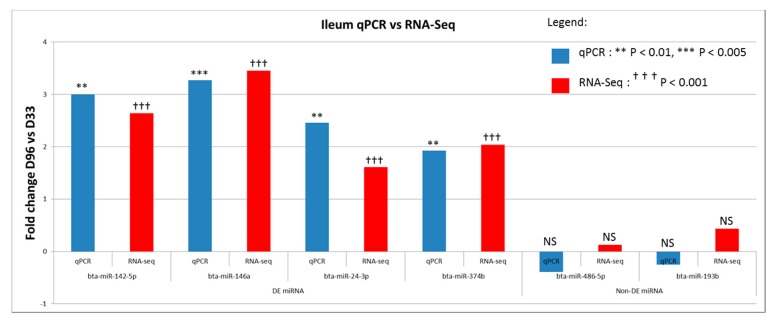
Result of qPCR validation of the expression of miRNAs between day 33 (pre-weaning) and day 96 (post-weaning), and compared with results obtained by miRNA sequencing.

The RNA-Seq expression of 6 miRNAs was validated by qPCR. Two of them (bta-miR-486-5p and bta-miR-193b) were non DE, while four of them (bta-miR-142-5p, miR-146a, miR-24-3p and miR-374b) were DE between D33 and D96 by RNA-Seq. Observed fold changes for DE miRNAs between both methods were similar, except for the non-DE miRNAs, where an opposite trend was observed (Figure 7).

## 4. Discussion

Physiologically, major metabolic changes that take place in calf gastrointestinal tract following the transition from liquid to solid food are accompanied by rapid changes in gene expression controlled by the signal-mediated coordination of transcriptional and post-transcriptional mechanisms [33]. Previously, we reported that ~20% of bovine genes were significantly DE between the pre-weaning (day 33) and the post-weaning (day 96) period, and enrichment analysis revealed the importance of DE genes in biological processes necessary for the switch in nutrition and developmental stage from the pre-weaning to the post-weaning period [23]. While it is well known that miRNAs are important for the regulation of these processes, little is known about how they participate in the regulation of ileum functions from the pre-weaning to the post-weaning period.

Highly expressed and DE miRNAs identified in this study suggest potential roles in ileum developmental processes during the transition from the pre-weaning to the post-weaning period. However, some highly expressed miRNAs such as bta-miR-21-5p, bta-miR-26a, bta-miR-148a and bta-let-7a-5p (Table 1) were also highly expressed in other tissues such as milk fat [10], milk whey/somatic cells [25], and mammary gland epithelial cells [13]. Interestingly, bta-miR-143, the most highly expressed miRNA, was also among the most abundant miRNAs in bovine testis [34] and reported as the most highly expressed miRNA in ileum tissue of calves at the pre- and post-weaning periods [35]. It was suggested that bta-miR-143 might regulate key genes involved in differentiation of connective tissue cells, the major components of the gut; hence, its high abundance might be important for the regulation of rapid development and growth of the gastrointestinal tract during early life. Indeed, enrichment analysis of target genes of the top 20 abundant miRNAs indicated enrichment in many biological processes and molecular function GO terms (Table 2 and Table S5) involved in developmental processes, therefore supporting important roles for highly abundant miRNAs, including bta-miR-143, in these processes. Further supporting evidence was derived from enriched KEGG pathways crucial for cellular development processes such as FoxO signaling pathway, cell cycle, MAPK signaling pathway, and p53 signaling pathway (Table 3). Moreover, we also detected 81 novel miRNAs in this study that were lowly expressed. The most abundant novel miRNA, bta-miR-22_24033, accounted for only 0.3% of the total read counts. Forty-four novel miRNAs were significantly DE between D33 and D96, therefore suggesting roles in the regulation of ileum gene expression during the early period of growth. Nevertheless, novel miRNAs identified in this study will enrich the bovine miRNome as well as enhance knowledge of the potential roles of miRNAs in calf GIT. However, further functional validations to clarify the roles of identified miRNAs in the development of calf gut during the early period of growth are needed.

Bta-miR-374a was the most significantly DE miRNA in this study (Table 4). Bta-miR-374a was found to be differentially expressed between lactating and non-lactating cows [36]. Bta-miR-374a potentially targeted 36 different genes (Table S6b) and some of them might be important for ileum functions such as *EIF2AK4*, *FBXO18*, *GTPBP3* and *GNB2*, etc. *EIF2AK4* is an important transcription factor in host response to infection with pathogenic bacteria associated with Crohn’s disease [37]. Moreover, *FBXO18*, *GTPBP3* and *GNB2* have been reported to be significantly DE in calf gastrointestinal tract between the pre- and post-weaning periods [23]. *FBXO18* encodes a member of the F-box protein family with function in phosphorylation-dependent ubiquitination [38]. *FBXO18* is implicated in the regulation of stress-induced apoptosis processes and homologous recombination in familial and sporadic breast cancer [39]. Cells deficient in *FBXO18* were unable to activate the cytotoxic-stress-induced cascade, resulting in increased cell survival [40]. *GTPBP3* is an important gene for mitochondrial functions and a mutation in this gene resulted in defective mitochondrial energy production through oxidative phosphorylation [41]. *GNB2* is important for neuronal apoptosis and was induced by lidocaine in the rat [42]. Nevertheless, the functions of these genes (*FBXO18*, *GTPBP3* and *GNB2*) in the ileum are unknown. Bta-miR-15b, the second most significantly up-regulated miRNA, belongs to the miR-15b family cluster. This miRNA cluster can target cell cycle proteins and the anti-apoptotic *Bcl-2* gene to control cell proliferation and apoptosis [43]. Bta-miR-15b might also play a role in mastitis disease development in cows [44]. Moreover, Liang et al. [35] also reported that bta-miR-15b was significantly DE between 0-day-old and 7-day-old calves and its expression correlated with bacterial population, thus suggesting roles in the regulation of gut development, immune, and digestive functions. Furthermore, bta-miR-15b potentially targeted *IKBKB* (Figure 6), a gene known as an essential molecule for NF-κB signaling pathway [45] with important roles in both innate and acquired immunity [46]. 

Interestingly, bta-miR-26a, one of the most significantly DE miRNA (Table 4), was also one of the most highly expressed miRNA (Table 1). The human homologue of this miRNA plays important roles in Crohn’s disease [15]. In cattle, this miRNA regulates the expression of *PCK1* gene, which is important for semen quality and longevity of Holstein bulls [47]. Bta-miR-26a potentially targeted *BID* gene and their expressions were significantly correlated in this study (Figure 6). *BID* is a pro-apoptotic member of the Bcl-2 protein family with roles in the regulation of apoptosis processes [46]. Therefore, bta-miR-26a might have roles in ileum development via targeting the *BID* gene. Bta-miR-455-5p was the most down-regulated miRNA between D33 and D96. Bta-miR-455-5p was reported to be important for the function of granulosa cells of subordinate and dominant follicles during the early luteal phase of the bovine estrous cycle [48]. In humans, this miRNA homologue down regulated *RAB18* gene in gastric cancer [49]. In fact, bta-miR-455-5p also potentially targeted *RAB18* gene in this study (Figure 6). 

Among the top 20 DE miRNAs in this study, bta-miR-142-3p, bta-miR-142-5p, bta-miR-191, bta-miR-146a, bta-miR-210 and bta-miR-424-5p were also found to be DE in calf ileum at the pre-weaning and weaning periods [35]. Some of these miRNAs have been reported to play important roles in immune functions. For example, bta-miR-146a inhibited the mRNA and protein expression levels of *TRAF6* gene and acted as a negative feedback regulator of bovine inflammation and innate immunity through down regulation of the TLR4/TRAF6/NF-κB pathway in bovine mammary epithelial cells [50]. Furthermore, bta-miR-142-5p was important for bovine alveolar macrophage response to *Mycobacterium bovis* infection [51].

As expected, the target genes of DE miRNAs were enriched in important biological process GO-terms related to metabolic processes (such as cellular macromolecule metabolic process, macromolecule metabolic process and regulation of metabolic process) (Table 5 and Figure 2) and molecular function GO-terms related to metabolism of the macromolecule compound (such as organic cyclic compound binding, heterocyclic compound binding, nucleic acid binding and protein binding) (Table 5 and Figure 4), thus suggesting roles in the regulation of these processes. Interestingly, the target genes of DE miRNAs were also enriched in GO-terms like transcription factor activity and sequence-specific DNA binding (FDR = 3.63 × 10^−3^) (Table 5), thus suggesting their importance in transcription factor activity. The interaction between miRNAs and transcription factors to regulate gene expression in biological processes is well documented [52,53]. MiRNAs might inhibit transcription factor activities by either directly inhibiting the expression of their encoding genes or by inhibiting other gene(s) that have impact on their activities [53,54]. Previously, we observed that some transcription factors might play important roles in mediating miRNA regulatory functions in cow milk yield and milk component traits [9]. In humans, several miRNAs have been reported to participate in the regulation of intestinal transcription factors; miR-196b inhibited the *GATA6* intestinal transcription factor to control intestinal cell homeostasis and tumorigenesis in colon cancer patients [55], while miR-30 family controlled intestinal epithelial cell proliferation and differentiation by targeting SOX9 (transcription factor) and other genes in ubiquitin ligase pathway [56]. In fact, as mentioned above, the most DE miRNA in this study (bta-miR-374) also potentially targeted a transcription factor (*EIF2AK4*) known to be important for human Crohn’s disease [37]. The most important pathway enriched for DE miRNAs target genes was Hedgehog signaling pathway (*p* = 0.003, Table S7, Figure 5). Hedgehog signaling pathway is important for cell growth, survival and fate, as well as for normal embryonic development [57,58]. This pathway also has multiple patterning functions during mammalian gut development [59]; therefore, it may be important for ileum functions during the early part of life. Another important pathway enriched for target genes of DE miRNAs was peroxisomes pathway (*p* = 0.006, Table S7, Figure 5). The peroxisomes pathway is crucial for metabolic processes such as fatty acid oxidation, biosynthesis of ether lipids, and free radical detoxification [60]. Since one of the main functions of the ileum is to absorb bile salts, one of the products of fatty acid oxidation, enrichment of the peroxisome pathway supports its role in normal ileum function. Another important role of the ileum is vitamin absorption and the enriched pathway, vitamin digestion and absorption (Table S7 and Figure 5), might reflect the changes in gene expression for different vitamin requirements between the pre- and post-weaning periods. Other enriched pathways also reflect the importance of miRNAs in the regulation of genes involved in lipid metabolism (phosphatidylinositol signaling system and sphingolipid signaling pathway), protein metabolism (valine, leucine and isoleucine biosynthesis and protein processing in endoplasmic reticulum) and intracellular signaling (cGMP-PKG signaling, FoxO signaling and neurotrophin signaling pathway) in the development of the ileum during the early part of life.

## 5. Conclusions

This is the first integrated miRNA-mRNA analysis characterizing the function of miRNAs in calf ileum during early life. Eighty-one novel miRNAs were identified that will enrich the bovine miRNome repertoire and contribute to the understanding of regulatory processes in calf ileum. This study highlighted potential roles of bta-miR-143, bta-miR-192, bta-miR-26a and bta-miR-21-5p in growth and developmental processes during the transition from the pre-weaning to the post-weaning period. This study also suggested roles for DE miRNAs in metabolic processes, metabolism of the macromolecule compound, transcription factor activities, as well as involvement in pathways related to metabolism (peroxisomes), vitamin digestion and absorption, lipid and protein metabolism, and intracellular signaling (Hedgehog signaling, GMP-PKG signaling, FoxO signaling, neurotrophin signaling pathway). Moreover, several DE miRNAs—DE mRNAs pairs such as bta-miR-374a—*FBXO18*, bta-miR-374a*—GTPBP3* and bta-miR-374a—*GNB2* with potential roles in tissue development, and bta-miR-15b—*IKBKB* with vital roles in immune functions were revealed. This study, therefore, provided insights on miRNA expression and their potential functions in calf ileum development during early life, which might facilitate identification of miRNA biomarkers for growth, nutritional and disease challenges during the pre- and post-weaning periods. 

## Figures and Tables

**Table 1:** The 20 most abundantly expressed miRNAs in ileum tissue of calves.

miRNA	Pre-Weaning (D33)		Post-Weaning (D96)		Both Periods	
	Read Counts	% of Total	Read Counts	% of Total	Read Counts	% of Total
bta-miR-143	16,742,092	24.51	7,468,034	13.65	24,210,126	19.68
bta-miR-192	4,435,941	6.50	5,383,934	9.84	9,819,875	7.98
bta-miR-26a	2,861,953	4.19	4,861,644	8.89	7,723,597	6.28
bta-miR-191	1,691,133	2.48	4,106,915	7.51	5,798,048	4.71
bta-miR-10b	1,693,467	2.48	2,031,077	3.71	3,724,544	3.03
bta-miR-148a	1,702,572	2.49	1,754,282	3.21	3,456,854	2.81
bta-miR-10a	1,413,716	2.07	1,734,580	3.17	3,148,296	2.56
bta-miR-21-5p	4,233,604	6.20	1,673,008	3.06	5,906,612	4.80
bta-miR-99a-5p	1,282,425	1.88	1,452,116	2.65	2,734,541	2.22
bta-miR-215	1,320,560	1.93	1,373,575	2.51	2,694,135	2.19
bta-miR-27b	1,557,472	2.28	1,310,625	2.40	2,868,097	2.33
bta-let-7a-5p	1,729,397	2.53	1,231,252	2.25	2,960,649	2.41
bta-let-7f	1,668,877	2.44	1,226,489	2.24	2,895,366	2.35
bta-miR-125b	923,543	1.35	987,775	1.81	1,911,318	1.55
bta-miR-145	1,152,495	1.69	798,715	1.46	1,951,210	1.59
bta-miR-30e-5p	699,838	1.02	735,220	1.34	1,435,058	1.17
bta-let-7g	1,083,909	1.59	685,389	1.25	1,769,298	1.44
bta-miR-194	1,498,650	2.19	571,343	1.04	2,069,993	1.68
bta-miR-30d	747,419	1.09	560,435	1.02	1,307,854	1.06

**Table 2:** Enriched gene ontology (GO) terms for target genes of 20 most abundantly expressed miRNAs.

GO Class	GOID	GO Term	*p*-Value	FDR
Biological process	GO:0044767	Single-organism developmental process	4.40 × 10^−13^	1.13 × 10^−10^
	GO:0044260	Cellular macromolecule metabolic process	7.96 × 10^−13^	1.79 × 10^−10^
	GO:0044237	Cellular metabolic process	3.29 × 10^−12^	6.57 × 10^−10^
	GO:0007275	Multicellular organismal development	2.62 × 10^−11^	4.71 × 10^−9^
	GO:0048731	System development	7.45 × 10^−11^	1.22 × 10^−8^
	GO:0009888	Tissue development	9.21 × 10^−11^	1.38 × 10^−8^
	GO:0048856	Anatomical structure development	1.24 × 10^−10^	1.72 × 10^−8^
	GO:0036211	Protein modification process	8.55 × 10^−10^	9.61 × 10^−8^
	GO:0030154	Cell differentiation	1.74 × 10^−9^	1.74 × 10^−7^
Cellular component	GO:0043412	Macromolecule modification	3.53 × 10^−9^	3.34 × 10^−7^
	GO:0005622	Intracellular	2.57 × 10^−20^	4.63 × 10^−17^
	GO:0044424	Intracellular part	1.35 × 10^−19^	1.22 × 10^−16^
	GO:0043227	Membrane-bounded organelle	3.43 × 10^−17^	2.06 × 10^−14^
	GO:0043231	Intracellular membrane-bounded organelle	1.95 × 10^−15^	8.79 × 10^−13^
	GO:0043229	Intracellular organelle	3.08 × 10^−15^	1.11 × 10^−12^
	GO:0005737	Cytoplasm	4.55 × 10^−15^	1.36 × 10^−12^
	GO:0044422	Organelle part	2.39 × 10^−10^	3.07 × 10^−8^
	GO:0044446	Intracellular organelle part	3.80 × 10^−10^	4.56 × 10^−8^
	GO:0044444	Cytoplasmic part	9.28 × 10^−10^	9.83 × 10^−8^
	GO:0005634	Nucleus	7.71 × 10^−8^	4.34 × 10^−6^
Molecular function	GO:0005515	Protein binding	3.18 × 10^−7^	1.10 × 10^−5^
	GO:0019207	Kinase regulator activity	4.91 × 10^−6^	1.08 × 10^−4^
	GO:0019887	Protein kinase regulator activity	3.01 × 10^−5^	4.79 × 10^−4^
	GO:0003723	RNA binding	3.24 × 10^−5^	5.06 × 10^−4^
	GO:0019210	Kinase inhibitor activity	3.78 × 10^−5^	5.76 × 10^−4^
	GO:0004702	Receptor signaling protein serine/threonine kinase activity	8.23 × 10^−5^	1.06 × 10^−3^
	GO:0005057	Receptor signaling protein activity	1.94 × 10^−4^	2.02 × 10^−3^
	GO:0061650	Ubiquitin-like protein conjugating enzyme activity	3.20 × 10^−4^	2.96 × 10^−3^
	GO:0003700	Transcription factor activity, sequence-specific DNA binding	4.99 × 10^−4^	4.17 × 10^−3^

**Table 3:** Enriched KEGG pathways for target genes of 20 most abundantly expressed miRNAs.

KEGG Pathway	*p*-Value	FDR
Cysteine and methionine metabolism	2.56 × 10^−3^	4.39 × 10^−2^
Amino sugar and nucleotide sugar metabolism	1.73 × 10^−3^	3.47 × 10^−2^
TGF-beta signaling pathway	9.58 × 10^−4^	2.30 × 10^−2^
Signaling pathways regulating pluripotency of stem cells	6.67 × 10^−4^	2.00 × 10^−2^
Pathways in cancer	3.81 × 10^−5^	4.57 × 10^−3^
Transcriptional misregulation in cancer	1.76 × 10^−3^	3.26 × 10^−2^
Proteoglycans in cancer	1.39 × 10^−4^	8.37 × 10^−3^
MAPK signaling pathway	1.22 × 10^−5^	2.94 × 10^−3^
Cell cycle	3.48 × 10^−4^	1.67 × 10^−2^
p53 signaling pathway	9.32 × 10^−4^	2.49 × 10^−2^
Protein processing in endoplasmic reticulum	1.29 × 10^−3^	2.81 × 10^−2^
ErbB signaling pathway	5.55 × 10^−4^	1.90 × 10^−2^
FoxO signaling pathway	1.04 × 10^−4^	8.35 × 10^−3^
Chronic myeloid leukemia	5.21 × 10^−4^	2.08 × 10^−2^

**Table 4:** The ten most up- and down-regulated miRNAs between D33 (pre-weaning) and D96 (post-weaning).

miRNA	Base Mean	Log2fold Change	Fold Change	*p*-Value	FDR
bta-miR-374a	8851.83	−1.59	−3.01	5.00 × 10^−29^	1.94 × 10^−26^
bta-miR-15b	17,755.36	−1.73	−3.32	7.96 × 10^−24^	1.31 × 10^−21^
bta-miR-26a	494,446.71	−1.32	−2.50	1.30 × 10^−20^	1.26 × 10^−18^
bta-miR-191	367,869.82	−1.81	−3.51	3.59 × 10^−18^	1.55 × 10^−16^
bta-miR-142-5p	92,116.08	−1.40	−2.64	6.64 × 10^−18^	2.58 × 10^−16^
bta-miR-146a	73,690.12	−1.79	−3.45	2.04 × 10^−16^	6.08 × 10^−15^
bta-miR-374b	4629.32	−1.03	−2.04	6.14 × 10^−16^	1.70 × 10^−14^
bta-miR-6119-5p	1796.83	−1.68	−3.19	2.08 × 10^−15^	5.38 × 10^−14^
bta-miR-340	360.85	−1.58	−2.99	1.40 × 10^−14^	3.31 × 10^−13^
bta-miR-361	9880.75	−1.39	−2.62	4.33 × 10^−13^	8.00 × 10^−12^
bta-miR-455-5p	282.27	1.89	3.70	1.01 × 10^−23^	1.31 × 10^−21^
bta-miR-210	486.48	1.67	3.19	4.23 × 10^−20^	3.28 × 10^−18^
bta-miR-497	692.69	1.70	3.24	9.95 × 10^−20^	6.43 × 10^−18^
bta-miR-103	39,682.96	1.00	2.00	1.69 × 10^−18^	9.35 × 10^−17^
bta-miR-142-3p	1742.08	1.52	2.87	1.96 × 10^−18^	9.52 × 10^−17^
bta-miR-3431	614.77	1.58	2.98	1.58 × 10^−17^	5.57 × 10^−16^
bta-miR-378c	1268.42	1.67	3.18	3.21 × 10^−17^	1.04 × 10^−15^
bta-miR-452	393.30	1.26	2.39	1.45 × 10^−14^	3.31 × 10^−13^
bta-miR-424-5p	301.96	1.90	3.73	1.84 × 10^−14^	3.97 × 10^−13^
bta-miR-326	378.11	1.49	2.82	6.07 × 10^−14^	1.24 × 10^−12^

**Table 5:** Most significantly enriched gene ontology (GO) terms for target genes of differentially expressed miRNAs.

GO Class	GO ID	GO Term	*p*-Value	FDR
Biological process	GO:0044260	Cellular macromolecule metabolic process	9.38 × 10^−10^	5.11 × 10^−7^
	GO:0043170	Macromolecule metabolic process	6.49 × 10^−10^	7.07 × 10^−7^
	GO:0019222	Regulation of metabolic process	9.86 × 10^−9^	3.58 × 10^−6^
	GO:0048518	Positive regulation of biological process	1.67 × 10^−8^	4.55 × 10^−6^
	GO:0071704	Organic substance metabolic process	4.25 × 10^−8^	7.72 × 10^−6^
	GO:0090304	Nucleic acid metabolic process	3.66 × 10^−8^	7.97 × 10^−6^
	GO:c0060255	Regulation of macromolecule metabolic process	9.84 × 10^−8^	1.34 × 10^−5^
	GO:0044237	Cellular metabolic process	8.67 × 10^−8^	1.35 × 10^−5^
	GO:0009059	Macromolecule biosynthetic process	1.76 × 10^−7^	1.60 × 10^−5^
	GO:0048522	Positive regulation of cellular process	1.92 × 10^−7^	1.61 × 10^−5^
Cellular component	GO:0005622	Intracellular	2.76 × 10^−21^	3.37 × 10^−19^
	GO:0043229	Intracellular organelle	2.39 × 10^−21^	5.85 × 10^−19^
	GO:0044424	Intracellular part	1.21 × 10^−20^	9.85 × 10^−19^
	GO:0043231	Intracellular membrane-bounded organelle	5.54 × 10^−18^	3.39 × 10^−16^
	GO:0043227	Membrane-bounded organelle	1.80 × 10^−17^	8.80 × 10^−16^
	GO:0005634	Nucleus	1.41 × 10^−12^	5.76 × 10^−11^
	GO:0005737	Cytoplasm	1.69 × 10^−10^	5.90 × 10^−9^
	GO:0005654	Nucleoplasm	1.80 × 10^−9^	5.52 × 10^−8^
	GO:0044428	Nuclear part	4.96 × 10^−9^	1.35 × 10^−7^
Molecular function	GO:0044446	Intracellular organelle part	1.06 × 10^−8^	2.59 × 10^−7^
	GO:0097159	Organic cyclic compound binding	1.19 × 10^−6^	1.19 × 10^−4^
	GO:1901363	Heterocyclic compound binding	6.87 × 10^−7^	1.37 × 10^−4^
	GO:0043167	Ion binding	2.32 × 10^−5^	1.16 × 10^−3^
	GO:0003676	Nucleic acid binding	2.03 × 10^−5^	1.36 × 10^−3^
	GO:0005515	Protein binding	3.90 × 10^−5^	1.56 × 10^−3^
	GO:0019207	Kinase regulator activity	5.59 × 10^−5^	1.60 × 10^−3^
	GO:0019887	Protein kinase regulator activity	5.31 × 10^−5^	1.77 × 10^−3^
	GO:0043169	Cation binding	1.39 × 10^−4^	3.47 × 10^−3^
	GO:0003700	Transcription factor activity, sequence-specific DNA binding	1.63 × 10^−4^	3.63 × 10^−3^
	GO:0019899	Enzyme binding	5.60 × 10^−4^	8.61 × 10^−3^

## Supplementary Materials

The following are available online at http://www.mdpi.com/2073-4409/7/9/134/s1. Table S1: Mapping statistics of miRNA sequencing reads; Table S2: Length distribution (nt) of miRNA reads; Table S3: Novel miRNAs identified by miRDeep2; Table S4: Novel and known miRNAs and their read count summary; Table S5; The 20 most highly expressed miRNAs and their predicted target genes (a); enriched gene ontology terms (b) and enriched KEGG pathways (c); Table S6; Differentially expressed miRNAs between day 33 (pre-weaning) and day 96 (post-weaning) (a); predicted target genes of DE miRNAs (b) and differentially expressed genes which are both negatively correlated and predicted targets of miRNAs (c); Table S7; Gene ontology and KEGG pathways enriched for target genes of differential expressed miRNAs; Table S8; Differentially expressed genes that were targets of DE miRNAs and also negatively correlated.
